# Supplementary material for: Novel Silyl Ether-Based Acid-Cleavable Antibody-MMAE Conjugates with Appropriate Stability and Efficacy
Source: Cancers (Basel). 2019 Jul 8;11(7):957. doi: 10.3390/cancers11070957 (PMC6678733; doi:10.3390/cancers11070957)
Supplement: Supplementary file 1 [file cancers-11-00957-s001.pdf]

# Supplementary Materials: Novel Silyl Ether-Based Acid-Cleavable Antibody-MMAE Conjugates with Appropriate Stability and Efficacy

**Yanming Wang, Shiyong Fan, Dian Xiao, Fei Xie, Wei Li, Wu Zhong and Xinbo Zhou**

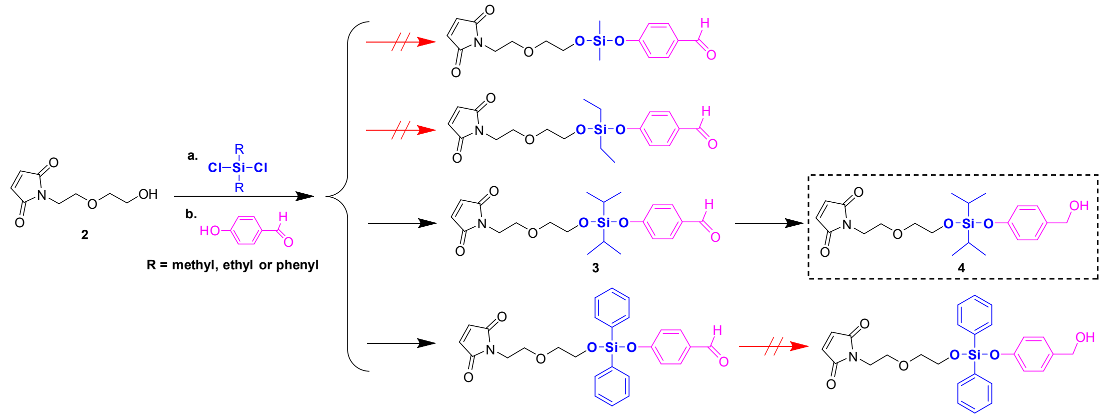


**Figure S1.** Synthesis and screening process of key intermediates of the designed silyl ether-based acid-cleavable linker.

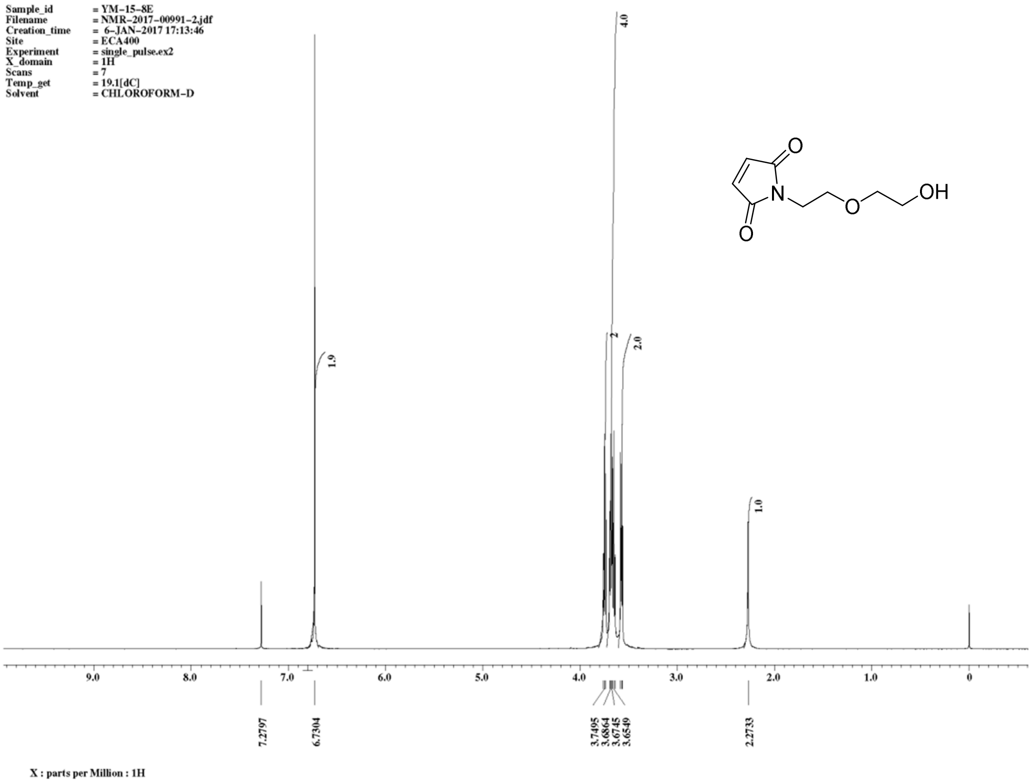


**Figure S2.** $^{1}$H-NMR spectrometry of compound 2.

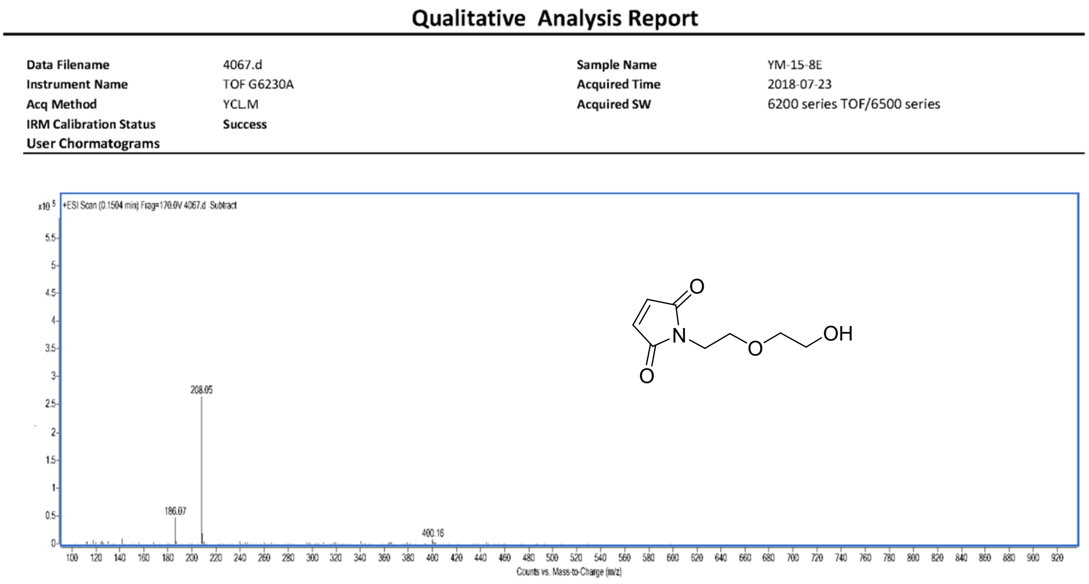


**Figure S3.** Mass spectrometry of compound 2.

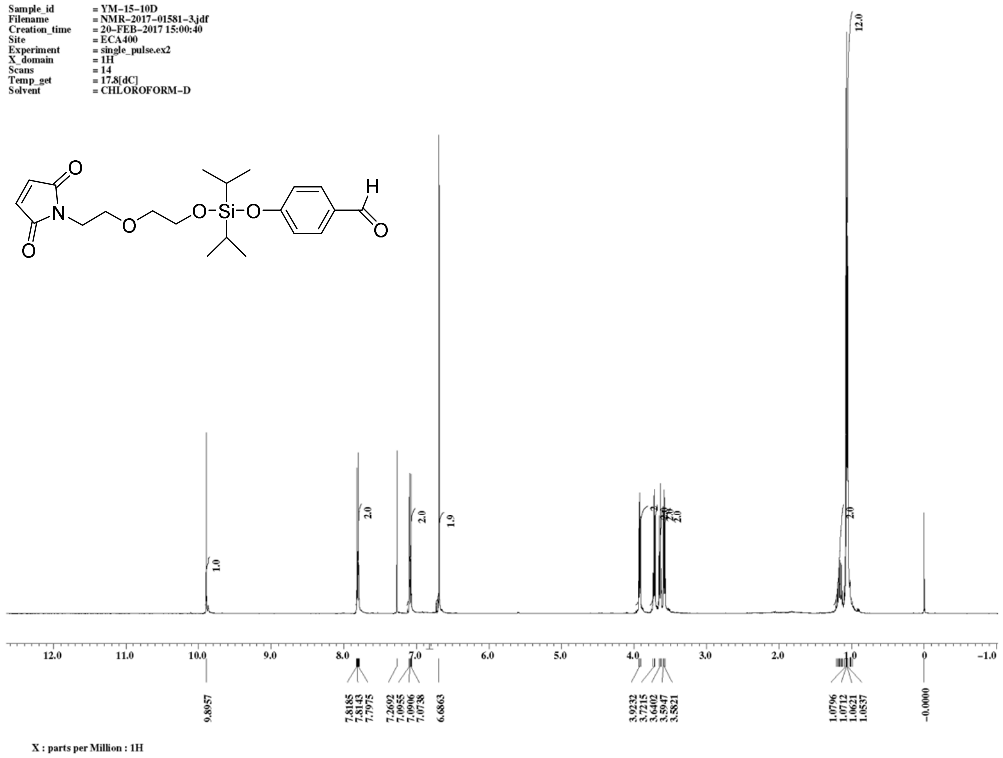


**Figure S4.** $^{1}$H-NMR spectrometry of compound 3.

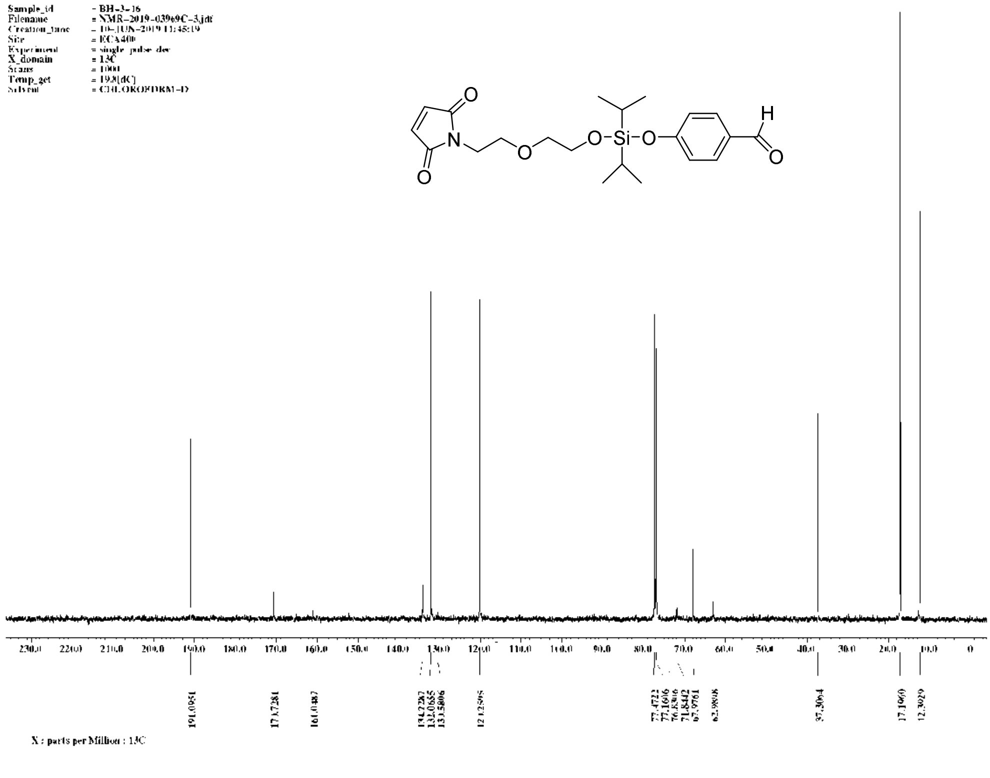


**Figure S5.** $^{13}$C-NMR spectrometry of compound 3.

## Qualitative Analysis Report

| | | | |
|---|---|---|---|
| **Data Filename** | 3954.d | **Sample Name** | BH-3-16 |
| **Instrument Name** | TOF G6230A | **Acquired Time** | 2019-06-10 |
| **Acq Method** | YCL.M | **Acquired SW** | 6200 series TOF/6500 series |
| **IRM Calibration Status** | Success | | |
| **User Chormatograms** | | | |

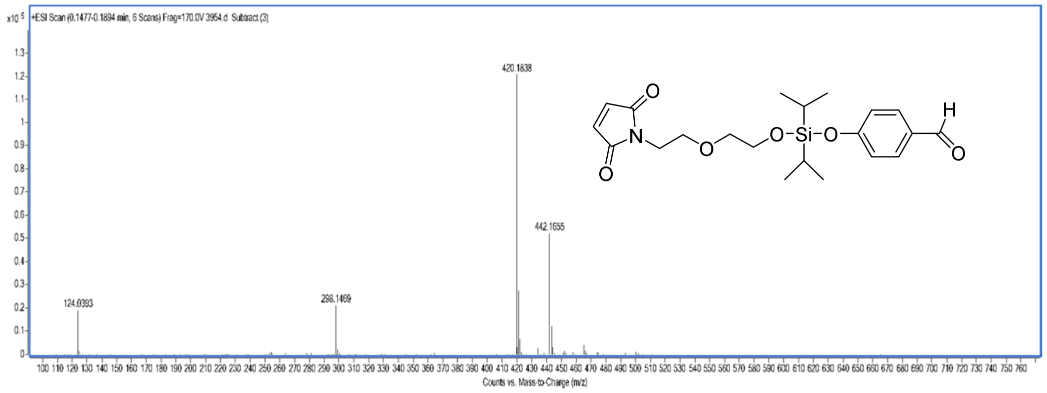


**Figure S6.** High resolution mass spectrometer of compound 3.

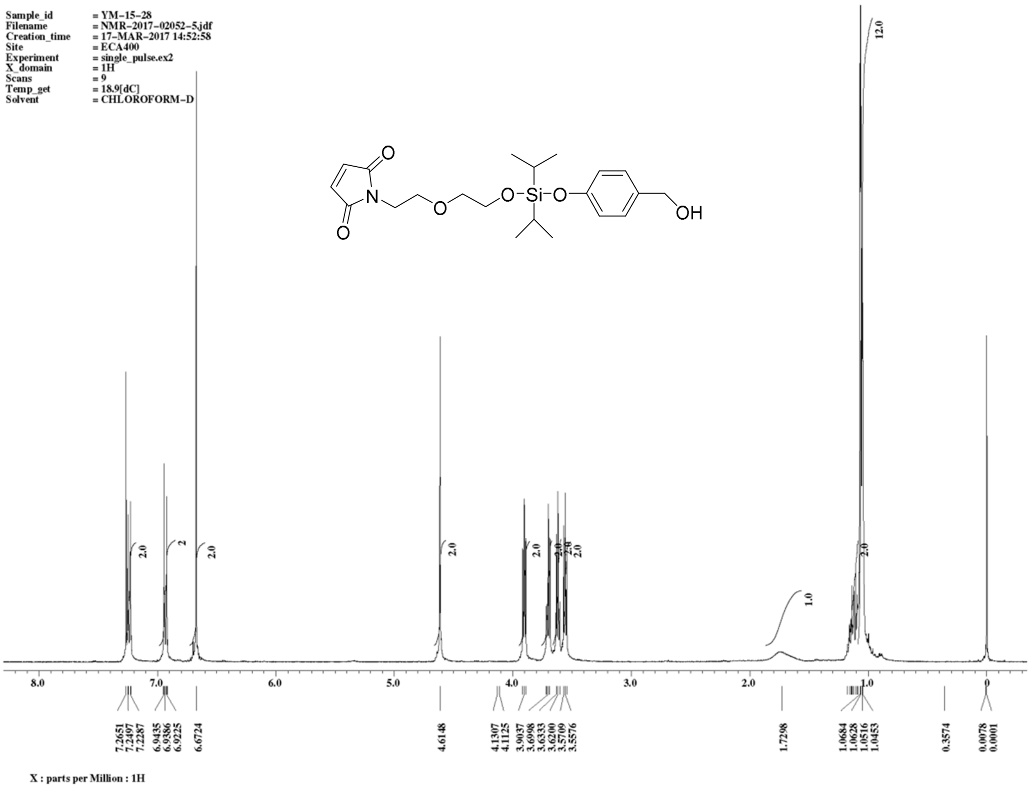


**Figure S7.** $^{1}$H-NMR spectrometry of compound 4.

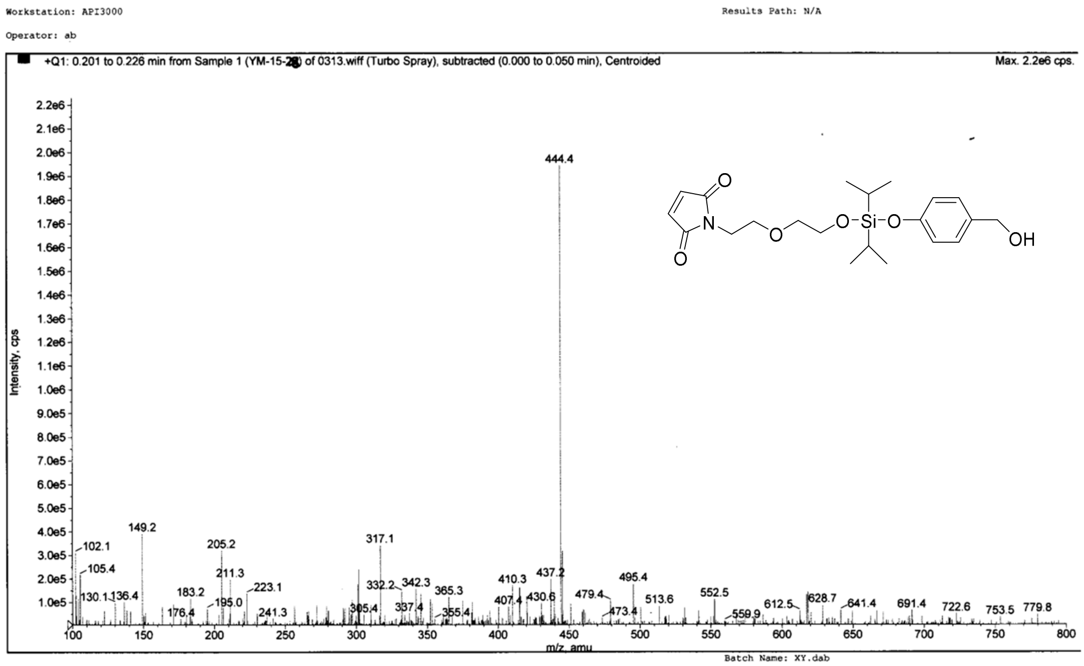


**Figure S8.** Mass spectrometry of compound 4.

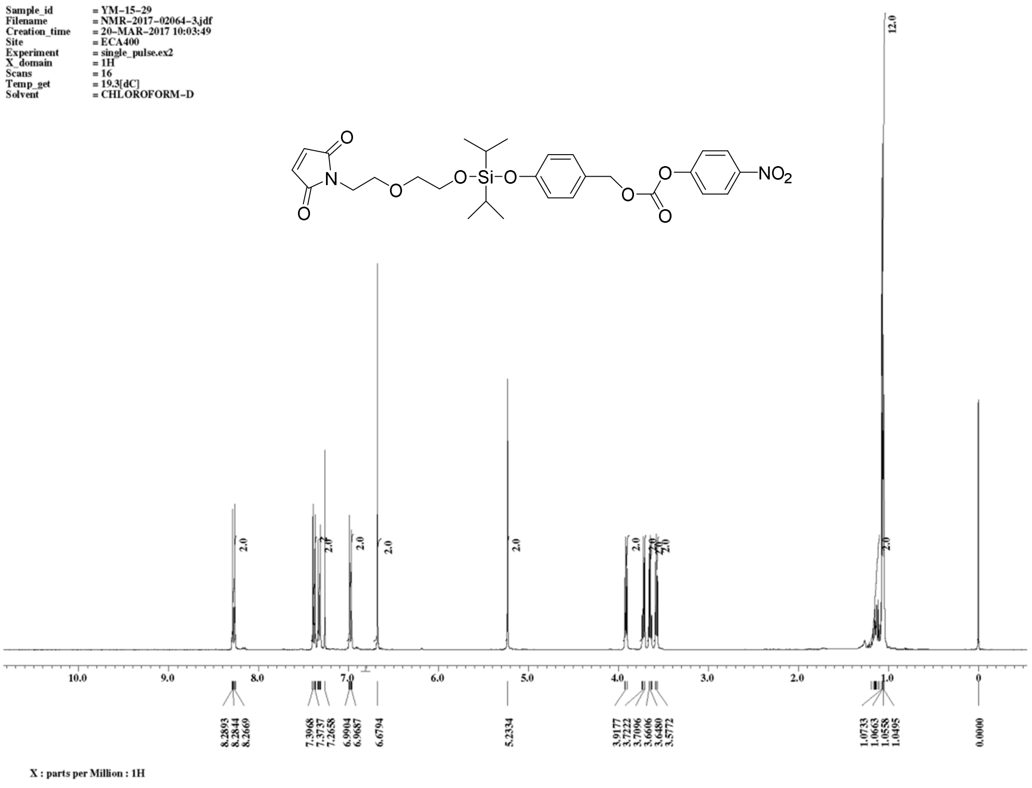


**Figure S9.** $^{1}$H-NMR spectrometry of compound 5.

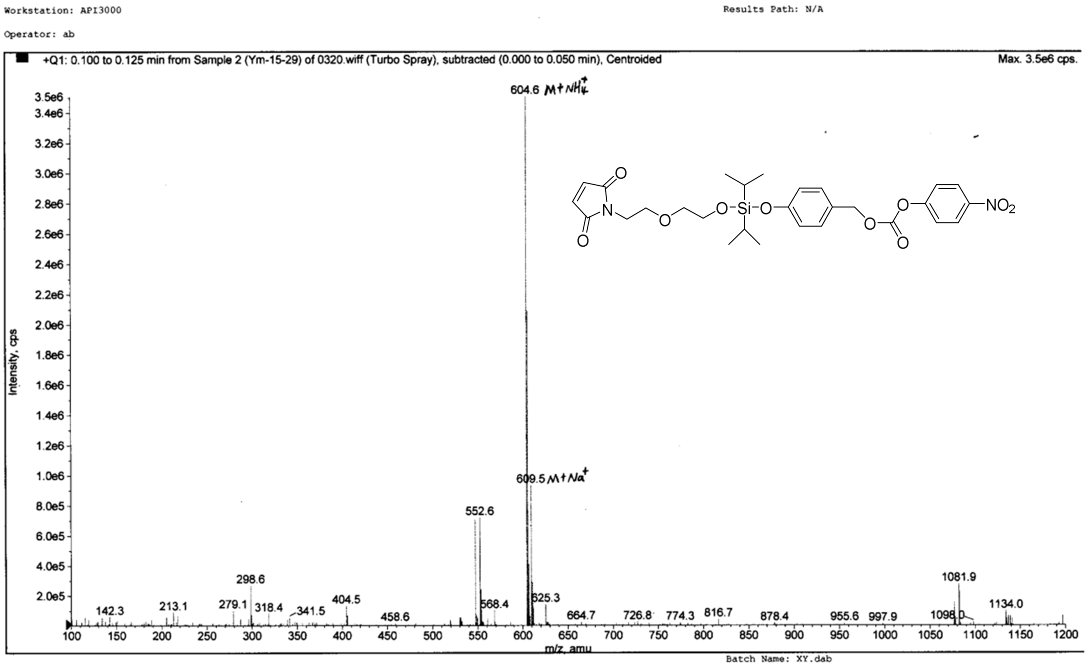


**Figure S10.** Mass spectrometry of compound 5.

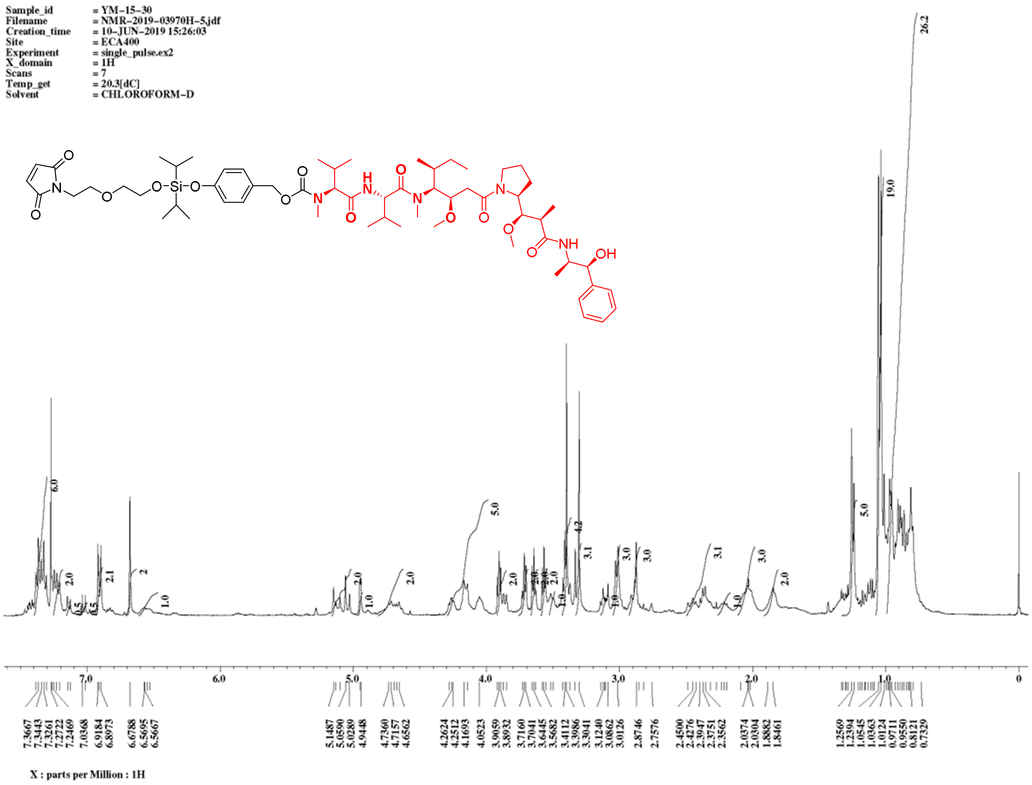


**Figure S11.** $^{1}$H-NMR spectrometry of compound 6.

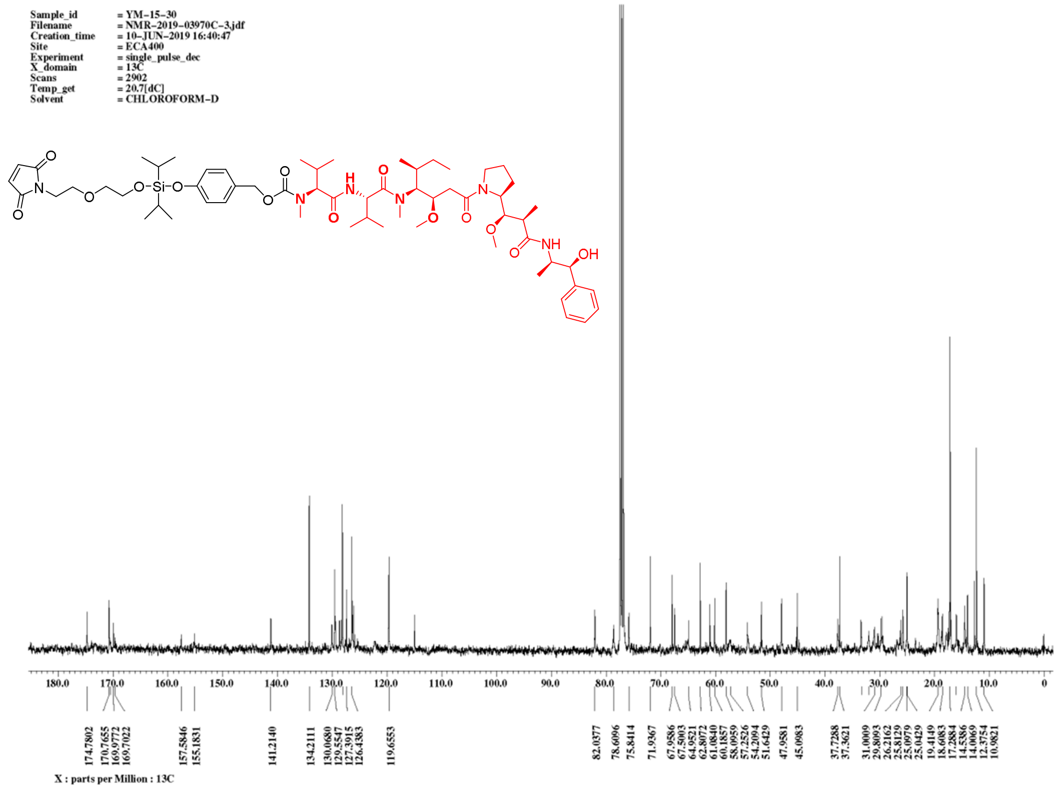


**Figure S12.** $^{13}$C-NMR spectrometry of compound 6.

## Qualitative Analysis Report

| | | | |
|---|---|---|---|
| **Data Filename** | 1291.d | **Sample Name** | YM-15-30 |
| **Instrument Name** | TOF G6230A | **Acquired Time** | 2017-03-22 |
| **Acq Method** | YCL.M | **Acquired SW** | 6200 series TOF/6500 series |
| **IRM Calibration Status** | **Success** | | |
| **User Chormatograms** | | | |

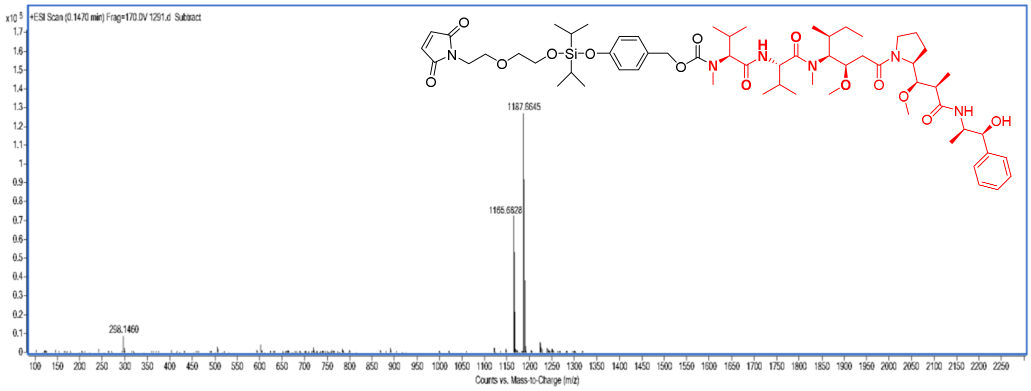


**Figure S13.** High resolution mass spectrometer of compound 6.

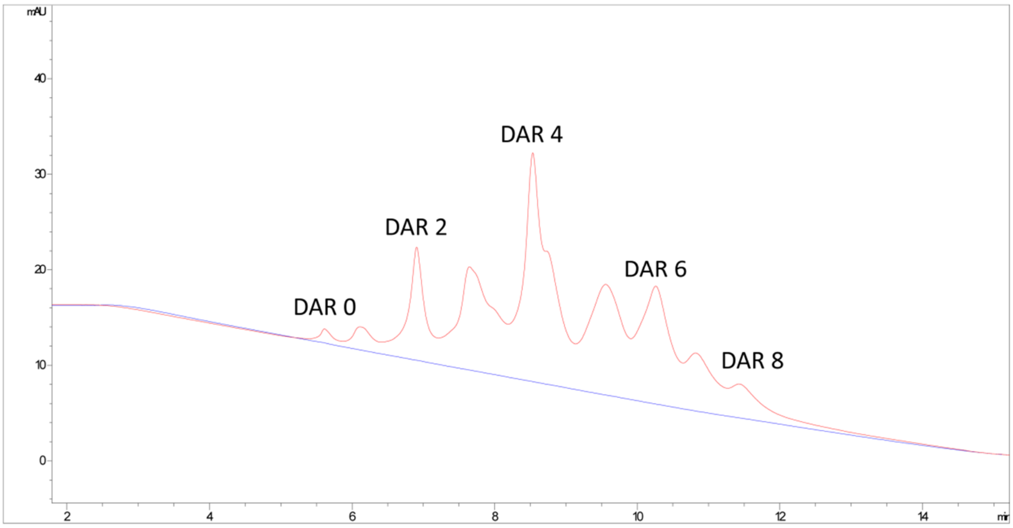


**Figure S14.** Hydrophobic interaction chromatography analysis of the ADC mil40-6 with an average DAR of about 5.5.
